# The Psychological Causes of Panic Buying Following a Health Crisis

**DOI:** 10.3390/ijerph17103513

**Published:** 2020-05-18

**Authors:** Kum Fai Yuen, Xueqin Wang, Fei Ma, Kevin X. Li

**Affiliations:** 1 School of Civil and Environmental Engineering, Nanyang Technological University, Singapore 639798, Singapore; kumfai.yuen@ntu.edu.sg; 2 Ocean College, Zhejiang University, Hangzhou 310058, China; kxli@zju.edu.cn; 3 School of Economics and Management, Chang’An University, Xi’an 710064, China; mafeixa@chd.edu.cn

**Keywords:** panic buying, purchasing behaviour, health crisis, determinants, COVID-19

## Abstract

Attributed to the recent COVID-19 pandemic, panic buying is now a frequent occurrence in many countries, leading to stockouts and supply chain disruptions. Consequently, it has received much attention from academics and the retail industry. The aim of this study is to review, identify, and synthesise the psychological causes of panic buying, which is a relatively new and unexplored area in consumer behaviour research. A systematic review of the related literature is conducted. The review suggests that panic buying is influenced by (1) individuals’ perception of the threat of the health crisis and scarcity of products; (2) fear of the unknown, which is caused by negative emotions and uncertainty; (3) coping behaviour, which views panic buying as a venue to relieve anxiety and regain control over the crisis; and (4) social psychological factors, which account for the influence of the social network of an individual. This study contributes to the literature by consolidating the scarce and scattered research on the causes of panic buying, drawing greater theoretical insights into each cause and also offers some implications for health professionals, policy makers, and retailers on implementing appropriate policies and strategies to manage panic buying. Recommendations for future research are also provided.

## 1. Introduction

In survival psychology, it is widely acknowledged that individuals may undergo behavioural changes following major events such as natural disasters and disease outbreaks that potentially disrupt social lives or even threaten individuals’ health [1]. One of such behavioural changes is panic buying, which occurs when consumers buy unusually large amounts of products in anticipation of, during or after a disaster or perceived disaster, or in anticipation of a large price increase or shortage.

Recently, panic buying was witnessed globally following the COVID-19 pandemic, and such behaviour was also observed and reported in past major disasters or health crises such as the 2011 Christchurch earthquake and 2016 Hurricane Mathew [2]. Panic buying is a socially undesirable, herd behaviour [3] where large quantities of daily necessities and medical supplies are purchased from markets, which often results in stockout situations. The situations limit or prevent individuals or more vulnerable groups (e.g., elderly or poor), who are in greater need of the products, from accessing them [4]. This generates negative externalities in societies. In addition, from the retail perspective, panic buying causes further disruptions to supply chains [5]. The sporadic surge in demand for consumer products, coupled with closures of routes or limits on traffic, poses challenges in areas like ordering, replenishment, and distribution. Consequently, this exacerbates stockout situations and often leads to price increase of consumer products.

Panic buying represents a relatively unexplored, niche area in consumer behaviour research where purchase decisions are impaired by emotions (e.g., fear of the unknown and anxiety) and social influences [6,7]. Hence, classical behavioural theories explaining consumer behaviour might not apply in this context. However, despite the importance of the topic, current understanding of panic buying is quite limited. A search on Scopus for academic materials that contain panic buying in their title, abstract, or keywords only yielded 32 results. Further, within these studies, only a few have directly examined the causes of panic buying. Most of the discussions on the causes of panic buying are very recent and are triggered by the COVID-19 pandemic. However, the discussions are chiefly based on academics, medical professionals, or reporters whose opinions and postulations are published in newspaper articles or online social media [8]. Overall, the findings or discussions are very scattered, focusing on a specific or a few causes that trigger panic buying. 

Due to the above observations, this study argues that it is now timely to consolidate the scarce and scattered literature on panic buying [9]. Hence, the objective of this study is to review, identify, and synthesise the causes of panic buying. This review study would provide a state-of-the-art understanding and assessment of panic buying, an agenda for future research, and interventions or policy recommendations to mitigate panic buying. The key principal findings of this study are that the causes of panic buying can be grouped into (i) perception, (ii) fear of the unknown, (iii) coping behaviour, and (iv) social psychological factors. Under the perception theme, perceived threat of the disease outbreak and perceived scarcity of products motivate panic buying. As for social psychological factors, they include social influence and social trust.

The rest of the study is organised as follows. First, it describes the methods used to extract and synthesise relevant journal articles, conference papers, and newspaper articles from search engines. Thereafter, the results are organised into themes and discussed individually. Finally, the implications and recommendations for future research are provided.

## 2. Methods

**Figure 1:**
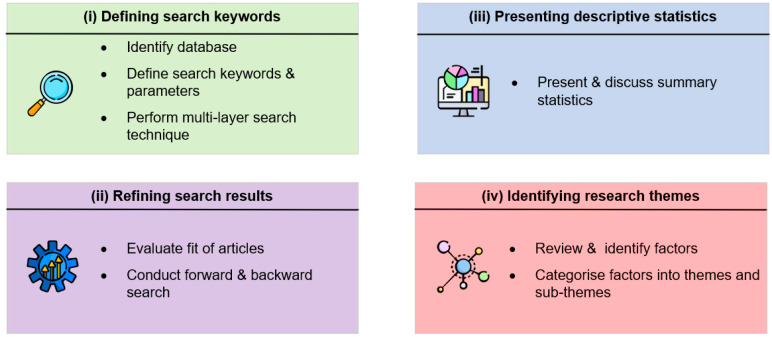
Overview of research method.

To reiterate, the objective of this study is to review, identify, and synthesise the psychological causes of panic buying. A systematic review of the literature adapted from Rowley and Slack [10] and Davarzani et al. [11] is conducted. Specifically, a four-stage method is employed. The four stages include (i) defining search keywords, (ii) refining search results, (iii) presenting descriptive statistics, and (iv) identifying research themes. Figure 1 summarises the key steps involved in each stage.

### 2.1. Defining Search Keywords

The first step of the research method involves identifying the database to conduct a search for related academic papers. Scopus is chosen because it is managed by the largest publishing house, Elsevier. Further, it is considered as the most comprehensive abstract and citation database, containing peer-reviewed literature in areas on technology, science, social sciences and arts and humanities, and medicine.

Thereafter, search keywords are defined (Table 1). Three groups or layers of keywords are developed (Table 1). Asterisk is used as the wildcard search to capture singular and plural forms, British and American English, and other verb or adjective forms of a keyword. For instance, “behavio*” would cover keywords such as “behaviour, “behaviour”, “behavioural”, “behaviours” and “behaviors”.

The first group consists of all keywords related to panic buying. A search of the literature that specifically mentions panic buying is very limited (*n* = 32). Hence, to further expand the search, other keywords that are considered imperfect substitutes of panic buying such as “stockpiling” and “hoarding” are used. Impulsive and compulsive buying are excluded from the search. Essentially, they are rather broad terms and do not connote similar meaning. Panic buying is a specific, herd behaviour that is mainly triggered by a disaster or health crisis. 

The second group contains keywords related to the causes of panic buying. Hence, all synonyms such as “cause”, “determinant”, “behaviour”, “influence” and “factor” are used in the search. The third group comprises keywords that are related to the context, which includes “disruption”, “disease”, “pandemic”, “consumption” or “purchase”. Due to rarity of the research on health crises, other extreme events such as natural disasters are also considered in the review.

After defining the keywords, a multi-layer search technique is performed. To improve the accuracy and relevance of the search, it is limited to the title, abstract, and keywords of articles. Further, the scope of the research is also limited to relevant subject areas on “business management and accounting”, “psychology”, “environmental sciences”, “social sciences”, “economics, econometrics and finance”, “arts and humanities”, and “multidisciplinary”. The search shall only cover the main types of academic literature (i.e., journal articles, conference papers, reviews, book chapters, and books). It is also limited to the literature published in English. 

The search was conducted on 15 March 2020. It begins with the first group of keywords. They are prioritised in the search because they are the most relevant group of keywords. Table 1 shows the search results before and after limiting the related subject areas. There are 4236 academic documents related to panic buying and its synonyms. Thereafter, the second-layer search, which includes keywords related to causes of panic buying, is performed. After limiting the subject areas, 2505 documents are found. Finally, a third-layer search is performed, which further restricts the search to the context. This reduces the number of documents to 277. This amount is deemed to be feasible for the authors to proceed to the next stage where manual screening of all the documents is required. 

### 2.2. Refining Search Results

The titles and abstracts of the 277 academic documents are reviewed for their relevance with the topic of this study. After the review, 18 documents are retained. The remainder has no bearings to the topic and is thus excluded. A large portion of the excluded documents relates to consumers’ hoarding and compulsive or impulsive purchasing behaviour. However, neither do they investigate the behaviour under the influence of a health crisis or natural disaster, which triggers panic buying, nor do they examine the causes of the behaviour. Another large portion of the excluded documents relates to industrial stockpiling, which focuses on managing safety stocks and locating warehouses for the optimal distribution of stocks or relief goods following a health crisis or natural disaster. These documents do not account for the purchasing behaviour of the public, which is central to the topic of this study.

A forward and backward search is subsequently conducted, which identifies and reviews the literature citing and cited by the 18 academic documents. At the end of the exercise, another 9 documents were found. All 27 documents are listed in chronological order in Appendix A.

### 2.3. Presenting Descriptive Statistics

The key characteristics of the 27 academic documents are presented in Table 2. It is noted that all documents are journal articles. According to Table 2, five of them are published in *The International Review of Retail, Distribution and Consumer Research* and two in the *International Journal of Disaster Risk Reduction*. The rest are published in different journals. To name a few, they include *International Journal of Environmental Research and Public Health* and *Sustainability*. The highlighted journals are potential avenues for submission of related manuscripts. 

Most of the journal articles are classified under the Business Management and Accounting (*f* = 16) and Economics, Econometrics and Finance categories (*f* = 8). A smaller number of journal articles are classified under the Psychology (*f* = 4), Decision Sciences (*f* = 3), Engineering (*f* = 3), Environmental Science (*f* = 3) and Social Sciences (*f* = 3) categories. 

Regarding the credibility of the journal articles, with the exception of one (i.e., SSRN), all are published by reputable academic journal publishers such as Taylor & Francis (*f* = 9), Elsevier (*f* = 6), and Wiley (*f* = 3). The journal publishers have a clear, robust peer review process that only publishes articles based on the recommendation of at least two independent reviewers and an editor. In addition, most articles (*f* = 25) are indexed by Clarivate Analytics and are awarded an impact factor which further highlights the credibility and quality of the articles. 

The number of articles related to panic buying has doubled since 2017, which suggests increasing attention towards consumer behaviour under extreme conditions, albeit the absolute number is minute. Further, it is observed that behavioural research on panic buying is only initiated in 2009. 

Regarding the method employed by the journal articles, about half (*f* = 14) has used correlational analysis. This includes employing multiple linear regression, logistics regression, or structural equation modelling to explore relationships between variables of interest. The second common method is to employ qualitative analysis such as interviews (*f* = 5) to draw deeper insights from their research questions. This is followed by using descriptive statistics such as presenting mean, standard deviation, rate of change and subgroup differences (*f* = 3), and optimisation which involves solving optimal inventories and locations (*f* = 3). The least common method is simulation, which examines the cascading impact of social networks on panic buying or the impact of panic buying on inventory management (*f* = 2).

### 2.4. Identifying Research Themes

The last step of the method involves reviewing the journal papers and identifying factors and theories that explain panic buying. First, the keywords found in each journal paper were identified and listed. Thereafter, the keywords that shared similar meaning were categorised under the same subtheme. Subsequently, the subthemes were organised under a broader theme where applicable. The aforementioned process was performed independently by the authors of this study. The outcomes were then compared, discussed, and modified. The details of the categorisation are shown in Appendix A. The summarised results are presented and discussed in the next section.

## 3. Results and Discussion

**Figure 2:**
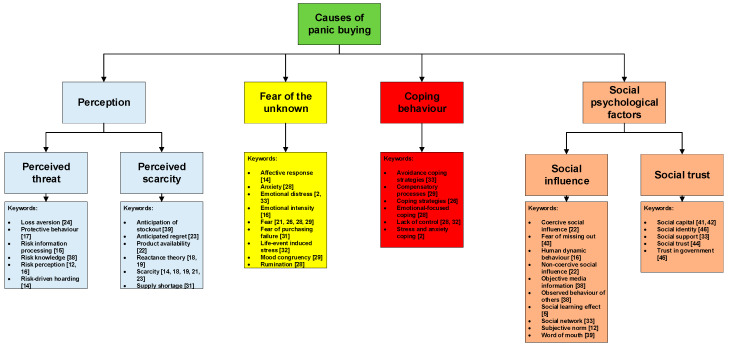
Themes, subthemes, and associated keywords.

This section presents and discusses the themes and subthemes that explain the causes of panic buying. As shown in Figure 2, the factors influencing panic buying can be categorised into four key themes: (1) perception, (2) fear of the unknown, (3) coping behaviour, and (4) social psychological factors. There are two sub-themes for perception: (1.1) perceived threat and (1.2) perceived scarcity. For social psychological factors, there are two sub-themes: (4.1) social influence and (4.2) social trust.

The theme with the highest mentions is individuals’ perception and assessment of the crisis (*f* = 15). This indicates that more than half of the 27 journal articles view panic buying to be triggered by individuals’ beliefs about the threat of an event, and product scarcity. The second highest concerns factors related to social psychological (*f* = 14). This suggests that approximately half of the journal articles concur that panic buying is a herd behaviour, which is influenced by the behaviour or dynamics of the crowd. The third is fear of the unknown, suggesting that panic buying is triggered by emotions and uncertainty (*f* = 10). Lastly, the least mentioned factor is coping behaviour (*f* = 6), proposing that panic buying is motivated by control deprivation. 

The subsequent subsections discuss the themes. In addition to the reviewed journal articles, recent newspaper articles which contain rich insights into the causes of panic buying due to the COVID-19 shall also be used to supplement the discussion. 

### 3.1. Perception

This theme covers individuals’ perception of a health crisis and other related events or entities that trigger panic buying. The review indicates two key dimensions. Accordingly, they are (i) perceived threat and (ii) perceived scarcity.

#### 3.1.1. Perceived Threat

During a health crisis, people form risk perception about the situation [12]. The degree of risk perceived by an individual is determined by his or her assessment of the threat of an outbreak, which can be measured by both the susceptibility and severity of the event [13]. Accordingly, susceptibility and severity refer to the probability and consequences of contracting a disease. 

Both susceptibility and severity are sub-dimensions of the health belief model, which posits that people are motivated to undertake self-protection behaviour to minimise risk. According to Sheu and Kuo [14], hoarding behaviour prior to or during a disaster, which can be viewed as a form of self-protection behaviour, is considered as a self-interested, planned behaviour in an attempt to minimise risk [15]. In particular, the risk can be hedged by storing large quantities of supplies which can confer individuals a sense of safety and well-being. For instance, by hoarding, individuals can reduce their visits to stores which minimises their contact with people, and hence, contracting the disease. Further, the stocks can serve as a safety buffer with the knowledge that there will be enough supplies (e.g., masks and hand sanitisers) to protect the hoarders and last through a health crisis [16].

According to Bish and Michie [17], there are three types of preventive behaviour that are performed by people in response to a health crisis. They include preventive, avoidant, and management of disease behaviours. Preventive behaviour includes maintaining hygiene by washing hands, wearing a mask, and cleaning surfaces. Avoidant behaviour includes minimising contact with others in crowds, public transport, or work. Management of disease behaviour relates to taking medication and seeking professional advice. All the activities associated with these three types of behaviour are expected to increase with the perceived threat of the health crisis. This will require more daily necessities or medical supplies to perform the behaviours, which triggers panic buying.

Therefore, in situations where the perceived threat of a disease is high, it is conceivable that an individual will be more likely to engage in panic buying to minimise the risk of contracting the disease. In this regard, panic buying can be viewed as a self-protection mechanism to satisfy the safety needs of individuals. 

#### 3.1.2. Perceived Scarcity

Perceived scarcity is strongly linked to reactance theory which posits that individuals experience psychological reactance, a motivational state that is about protecting their behavioural freedom when they feel threatened or restricted [18,19]. 

In this context, a product that is expected to become inaccessible soon due to a health crisis is likely to threaten or restrict personal freedom (i.e., prevented or reduced access to the product) [14,20]. Consequently, such signals would stimulate psychological reactance that raises the attention towards and attractiveness of a product. Psychological reactance can trigger a sense of urgency to buy and hoarding behaviour, which connote similar meaning with panic buying [21,22].

Another theory that links perceived scarcity with panic buying is anticipated regret [23]. This anticipated emotion manifests from a rejected option. For instance, people would compare their actual decision of hoarding with a forgone decision of not hoarding during the initial disease outbreak. Regret would be evoked if the rejected choice turns out to be better than the actual outcome whereas rejoice would be experienced if the actual outcome is better than the rejected choice. According to previous studies, these emotional consequences are anticipated and considered when making choices under uncertain situations. Consistent with prospect theory, during disease outbreak, it is more likely that people would experience regret than rejoice for not engaging in panic buying due to perceived scarcity [24].

Therefore, due to the above reasons, perceived scarcity of goods would motivate individuals to engage in panic buying due to psychological reactance and anticipated regret. 

### 3.2. Fear of the Unknown

In general, people would experience emotional distress such as fear and anxiety during a disease outbreak [25,26]. This distress is mainly caused by the inability of humans to predict the outcomes of an outbreak, which challenges the human dominance of nature [21]. Fear of the unknown is influenced by the lack of knowledge about a health crisis or disease. It creates uncertainty, which causes people to ruminate and imagine multiple scenarios and hence arouses fear [27,28]. 

Very often, fear is noted to modify shopping behaviour rather than the occurrence of the outbreak itself [29]. Past statistics suggest that spending at retail outlets increased significantly in preparation for impending disasters to prepare for the unknown [30,31]. 

According to Sneath et al. [32] and Kennett-Hensel et al. [33], fear motivates individuals to make purchases because that would confer them a sense of security, comfort, momentarily escape, and alleviate stress. Such motivation is often not driven by the actual need for the purchased products but rather an avenue for individuals to regulate their negative emotions. 

The correlation between fear and increased purchase behaviour could also be explained by mood congruency. It proposes that under negative emotions or stress, an individual’s perception and judgement of tangential situations or events are negatively skewed [29]. Heightened fear augments people’s perceived risk and threat of the situation [34,35], motivating them to take drastic measures to respond to a dramatic event such as a disease outbreak. Consequently, based on the discussion in Section 3.1.1, this can trigger panic buying, which is viewed as a form of self-protection behaviour to minimise their risk.

### 3.3. Coping Behaviour

Expanding from the discussion of Section 3.2, a stream of literature suggests that stressors such as fear of the unknown trigger coping behaviour [2,32]. In this regard, panic buying can be viewed as an outlet to regain control over the situation, which compensates for the psychological losses experienced by individuals [26]. 

Control refers to the ability to influence outcomes in one’s environment [36]. In general, humans have an innate desire to control, and it has a part to play in their survival [28]. There are many situations such as a health crisis that would reduce an individual’s perception of control over the environment. Consequently, this would cause discomfort which triggers the individual to regain control. According to compensatory control theory, when the source of discomfort (i.e., disease outbreak) is not amenable to control, the individual will turn to increase control over other domains. 

Individuals can exert control over the environment through problem solving. Existing studies show that engaging in problem solving reinforces individuals’ belief of regaining control over the situation. Very often, an individual engages in problem solving to transit from a situation (i.e., loss of control) that is less desirable to another that is more desirable (i.e., regain control). 

Essentially, there are two requirements that must be met for an individual to be motivated to perform problem solving [8]. First, the action must be executable by the individual. Second, the action must be believed to result in a more desirable state. In this regard, buying consumer products, especially daily necessities, meets the first requirement because it is an activity that is performed by households on a regular basis and does not consume much cognitive (i.e., effort to deliberate) or monetary resources. Further, it also meets the second requirement because buying consumer products is practical. Although buying large quantities of consumer goods is maladaptive [33] because it does not help or might even worsen the shortage of supplies in the market, it confers individuals with indirect control over the situation, knowing that most of these goods can help tide over the health crisis or can still be used in the future. 

To summarise, panic buying can be viewed as a compensatory consumption behaviour, which suggests that individuals turn to purchasing products as a way to restore deficits triggered by perceived needs and desires that can only be fulfilled indirectly [37]. In this regard, the deficit refers to the loss of control over the situation, and this can be compensated through problem solving such as panic buying. 

### 3.4. Social Psychological Factors

This theme reviews factors that cause the spread of panic buying through social networks. The two key factors are social influence and social trust.

#### 3.4.1. Social Influence

Individuals are members of a society. Therefore, their decisions can be influenced by the attitudes, opinions, and beliefs of the larger group [24]. Social influence refers to the way in which individuals adjust their behaviour to meet the demands of a social environment. There are several types of social influence that could explain panic buying.

The first type of social influence is self-fulfilling prophecy [8]. In this digital era, information is readily available and can be quickly disseminated to masses in multiple channels (e.g., social media, online news, radio, and chat applications). While these channels can facilitate the government or other health organisations to provide updates or advice to the public to cope with a health crisis, these channels are also susceptible to abuse. Misinformation and the spread of rumours such as stockout situations can influence individuals, either by fear of missing out or confusion into panic buying [38]. This reaction ultimately fulfils the once-false prophecy. 

Another type of social influence is normative influence [39]. It refers to the influence of others that leads to the conformance of a behaviour in order to be accepted by them. Normative influence can be exerted through peer pressure through word-of-mouth [39]. For instance, the constant advice from significant referents to stockpile can lead to conformance, driven by the need to identify and gain peer acceptance. 

The last type of social influence concerns observational learning [14], which is linked to the concept of information cascade that is rooted in behavioural economics and network theory [40]. It describes a phenomenon where people make the same decision in a sequential manner, resulting in a herd or crowd behaviour. As decisions (i.e., panic buy) are made sequentially, an individual has the opportunity to observe the choices made by those who acted earlier [5,38]. Since the individual can only observe but does not know the outside information (e.g., news and motivations) that others might possess, the individual will have to make inferences about the information that the others know. This lack of information may influence the individual to imitate the majority who are panic buying, overriding his or her own decision, believing that the majority has a better assessment of the situation and that panic buying is the optimum choice. 

#### 3.4.2. Social Trust

In addition to physical and human capital, Joshi and Aoki [41] highlighted the importance of social capital, which is defined as “social networks, norms, and social trust that facilitate mutual coordination and cooperation” [42] for disaster recovery programmes. 

There are two key aspects of social trust in times of crisis: trust in the community and government. Regarding trust in the community, it reflects the collectivistic nature of an individual, which is associated with attributes such as generosity, dependability, helpfulness, and attentiveness to others’ needs [40]. On the contrary, the individualistic nature is associated with attributes such as independence or assertiveness. The portrayal of people engaging in panic buying by the media can potentially cause distrust and subsequently triggers panic buying [43].

Regarding trust in the government, the government plays a crucial role in providing relief and recovery, maintaining order and control, and disseminating information to the public during a disease outbreak [44,45]. Public trust towards the government is crucial because that would ensure compliance and a concerted and coordinated effort to manage and control the spread of a disease [46]. 

To summarise, a high level of social trust would indicate that individuals would be more cooperative and considerate by not hoarding and sharing limited supplies with others [47]. Conversely, a high level of social distrust could cause the public to act individualistically, fearing others to buy more than their share and leaving none for others [48]. This triggers panic buying. 

## 4. Conclusions

In this section, a summary of the study, its contributions, and recommendations for future research studies will be provided.

This study has examined the existing state of research, summarised, categorised, and expanded current conceptual understandings of the psychological causes of panic buying. The review of the literature shows that the causes of panic buying can be categorised into four main themes: (1) individuals’ perception of the threat of a crisis and the scarcity of products, (2) fear of the unknown which is caused by emotions and uncertainty, (3) coping behaviour which is triggered by control deprivation, and (4) social psychological factors which consider the purchasing behaviour and dynamics of an individual’s social network.

The main contributions of this study are that it makes the first attempt to provide a more holistic explanation of the causes of panic buying. It consolidates the limited and scattered literature and organises them into a framework consisting of themes and sub-themes. Next, this study incorporates theories into the review, which draw greater academic connections between panic buying and the broader literature on consumer behaviour. It also summarises and describes the methods used by existing research and provides guidance for future researchers on choosing the suitable method for their study. For instance, future research that aims to explore the causes of panic buying can use qualitative methods such as conducting interviews or focus groups. Research that primarily focuses on quantifying the factors’ influence on panic buying could use a combination of descriptive statistics and correlational analysis such as regression analysis or structural equation modelling, after collecting observational or experimental survey data from the public. Research that aims to determine the optimal level of supplies or optimal locations for distribution of the supplies in anticipation of panic buying can employ optimisation methods. Lastly, research that analyses the spread or diffusion of panic buying within a society can employ simulation methods.

In addition, by understanding the psychology and motivations of panic buying, this study provides some implications for health professionals, policy makers, and retailers on implementing appropriate policies and strategies to curb panic buying. For instance, individuals’ perceived scarcity of products can be reduced by ensuring consistent assurance and messages from the government, media, and retailers that there is availability of stocks. Other signals of scarcity such as empty shelves and long queues could be mitigated by incentivising online delivery and implementing fast replenishment. Implementing appropriate sanctions and purchase quota on necessities could also prevent stockouts and consequently improve the public’s perception of products’ scarcity. This would also reduce the impact of information cascade. Next, any rumour and misinformation should also be immediately stopped and clarified by the media or the government to reduce uncertainty and fear and improve social trust. The media or government can also highlight the negative consequences of panic buying and persuade the public to be more generous by purchasing responsibly.

Finally, by consolidating the literature, this study alludes to avenues and opportunities for future research. This study recommends additional research to identify and examine the causes of panic buying using theories. The current theoretical understanding of the causes of panic buying is still quite limited. Hence, uncovering more causes and simultaneously estimating their influence on panic buying would provide empirical evidence and allow policy makers or retailers to focus on addressing the key determinants of panic buying. Further, additional research is proposed to be targeted at understanding the nomological structure of the causes of panic buying. With the use of theories, future research is recommended to examine the interrelationships of the causes of panic buying and identify potential mediators and moderators of these relationships. This would enrich existing knowledge of individuals’ cognitive process leading to panic buying. Finally, further research can also focus on examining the effectiveness of available interventions (e.g., implementing purchase quota and increasing product prices) or policies (e.g., sanctions or communication strategies) in curbing panic buying. The use of factorial experimental design or simulation would be useful for this purpose. Factorial experimental design is a method commonly used by psychologists to evaluate whether there is a link between variables. In this context, it subjects participants to various treatments, scenarios, or policy interventions to determine participants’ intention to engage in panic buying following a health crisis. Consequently, the influence of the treatments, scenarios, or policy interventions on panic buying can be determined and hence informs researchers and policy makers about their effectiveness.

There are a few limitations in this study. First, it only provides a current snapshot of the causes of panic buying based on the related literature. However, the literature is very limited. Therefore, future research is encouraged to explore other causes of panic buying that are not covered by this study. Another limitation is that this study is unable to determine the influence of each theme on panic buying. Future research can provide empirical evidence through conducting surveys and correlational analysis on the obtained data. 

## Figures and Tables

**Table 1:** Search keywords and results.

Search Keywords	Search Results ^1^	
	Before	After
(a) First-layer search structure	9397	4236
Panic buy* OR stockpile* OR hoard*		
(b) Second-layer search structure	5647	2505
Panic buy* OR stockpile* OR hoard*		
AND		
Cause* OR determin* OR behavior* OR influenc* OR factor* OR impact* OR affect* OR effect*		
(c) Third-layer search structure	769	277
Panic buy* OR stockpile*		
AND		
Cause* OR determin* OR behavior* OR influenc* OR factor* OR impact* OR affect* OR effect*		
AND		
Disrupt* OR disaster* OR disease* OR earthquake* OR pandemic OR outbreak* OR tsunami OR health* OR consum* OR purchas*		

^1^ Number of articles before and after limiting search to related subject areas.

**Table 2:** Descriptive statistics (*n* = 27).

Characteristics	*f*
Year of publication	
2020 (as of 15 March 2020)	3
2019	6
2018	4
2017	2
2016	2
2015	2
2014	2
2013	1
2012	1
2011	2
2010	1
2009	1
Journal title	
The International Review of Retail, Distribution and Consumer Research	5
International Journal of Disaster Risk Reduction	2
Others ^1^	20
Journal article category	
Business Management and Accounting	16
Economics, Econometrics and Finance	8
Psychology	4
Decision Sciences	3
Engineering	3
Environmental Science	3
Social Sciences	3
Others ^1^ (i.e., Energy, Medicine, Multidisciplinary & Public Administration)	4
Publisher	
Taylor & Francis	9
Elsevier	6
Wiley	3
MDPI	2
Springer	2
Others ^1^ (i.e., Emerald, Frontiers, Hindawi, IEEE & SSRN)	5
Impact factor (Clarivate Analytics)	
Yes	25
No	2
Peer reviewed	
Yes	26
No	1
Method	
Correlational analysis	14
Descriptive statistics	3
Interview	5
Optimisation	3
Simulation	2

^1^ Sub-characteristics with a frequency of one are aggregated.

## Appendix A

**Table A1:** Themes of reviewed literature.

Literature ^1^	Perception		Fear of the Unknown	Coping Behaviour	Social Psychological Factors	
	Perceived Threat	Perceived Scarcity			Social Influence	Social Trust
Kang, He, and Shin [43]					√	
Pan, Dresner, Mantin, and Zhang [22]		√				
Sheu and Kuo [14]	√	√	√		√	
Zheng, Shou, and Yang [5]					√	
Aliperti and Cruz [15]	√					
Gupta and Gentry [23]		√				
Wang, Liu, and Zhang [24]	√					
Wen, Sun, Li, He, and Tsai [12]	√				√	
Drury [46]						√
Kang and Skidmore [44]						√
Larson and Shin [29]			√	√		
Yoon, Narasimhan, and Kim [31]		√	√			
Forbes [2]			√	√		
Gao and Liu [16]	√		√		√	
Frank and Schvaneveldt [38]	√				√	
Gupta and Gentry [18]		√				
Sterman and Dogan [21]		√	√			
Yangui and Hajtaïeb El Aoud [39]		√			√	
Joshi and Aoki [41]						√
Kemp, Kennett-Hensel, and Williams [28]			√	√		
Thomas and Mora [19]		√				
Ballantine, Zafar, and Parsons [26]			√	√		
Kennett-Hensel, Sneath, and Lacey [33]			√	√	√	√
Aldrich [42]						√
Han, Hu, and Nigg [45]						√
Bish and Michie [17]	√					
Sneath, Lacey, and Kennett-Hensel [32]			√	√		
**Frequency (*f*)**	7	8	10	6	8	6

^1^ Cited literature is sorted in chronological order.
